# Recent advances in the induction of labor

**DOI:** 10.12688/f1000research.17587.1

**Published:** 2019-10-30

**Authors:** Anna Maria Marconi

**Affiliations:** 1 Department of Health Sciences, San Paolo Hospital Medical School, University of Milano, Milano, Italy

**Keywords:** Induction of labor, prostaglandins, prediction of successful induction

## Abstract

The rate of labor induction is steadily increasing and, in industrialized countries, approximately one out of four pregnant women has their labor induced. Induction of labor should be considered when the benefits of prompt vaginal delivery outweigh the maternal and/or fetal risks of waiting for the spontaneous onset of labor. However, this procedure is not free of risks, which include an increase in operative vaginal or caesarean delivery and excessive uterine activity with risk of fetal heart rate abnormalities. A search for “Induction of Labor” retrieves more than 18,000 citations from 1844 to the present day. The aim of this review is to summarize the controversies concerning the indications, the methods, and the tools for evaluating the success of the procedure, with an emphasis on the scientific evidence behind each.

## Introduction

Induction of labor (IOL) is certainly one of the most frequently performed obstetric procedures in the world: recent data indicate a percentage of induction of up to 35.5% in Sri Lanka ^1^, 24.5% in the United States ^2^, and from 6.8 to 33% in Europe ^3^. In spite of the extreme diffusion of the procedure, there are still numerous unanswered questions, or questions that have not obtained a unanimous consensus in the scientific literature. In general, it is universally accepted that IOL is indicated when it is thought that the outcomes for the fetus, the mother, or both are better than with expectant management, that is waiting for the spontaneous onset of labor ^4– 7^; in addition, IOL should be taken into consideration when the vaginal route is thought to be the most appropriate for delivery, a concept that is broader than the simple absence of contraindications to vaginal birth. Furthermore, being a medical procedure, IOL should be carried out only when there is informed consent ^4, 5, 8^ and where the precursor for the induction, including specific risks and benefits and the choice of the method used, are clearly explained; furthermore, I personally believe that consent should be accompanied by data on the success of the procedure in the birth center. A general concern is that IOL might increase the rate of cesarean delivery and have an impact on the experience of birth, as in women undergoing IOL it is generally less favorable, even though it is not always easy to separate the effect of the procedure *per se* from that of the perception of the obstetric risk that makes IOL necessary or from its outcome. In other words, the mother can perceive childbirth complicated by a risk that makes IOL necessary or that ends with a caesarean section as negative or less positive. When it comes to IOL, the factors to be taken into consideration and that can influence its success are many: among them, the precursor to induction, i.e. the clinical condition, present or absent, at the time the decision to induce is taken, the woman’s characteristics, the method of induction used, and other factors that can predict the success of the induction. However, it should be borne in mind that the current literature is not unanimous in defining certain key points such as the definition for failed induction or even what to consider as the success of the induction. We will address these points individually.

## Precursors for induction

Table 1 presents a summary of the five guidelines available on the subject ^4– 8^. The first line presents a general summary of the precursors with some guidelines being specific of some clinical situations while others remain more generic. In the case of the American College of Obstetricians and Gynecologists (ACOG), for instance, this vagueness is compensated for by the presence of a series of publications referring to specific clinical situations ^9– 13^, in which the possibility of induction is dealt with. Given the generality of the concept that IOL is indicated when terminating pregnancy is better than expectant management, I have then chosen to insert a series of specific precursors, those reported by the National Institute for Health and Care Excellence (NICE), the oldest of the guidelines, and then verify possible changes over time in the more recent.

**Table 1.** Summary of the available guidelines on the induction of labor.

	NICE, 2008 ^4^	ACOG, 2009 ^6^	WHO, 2011 ^7^	SOGC, 2013 ^8^	Queensland, 2017 ^5^
Summary of indications	10 specific circumstances	Possible indications	Five specific circumstances	High priority Other indications Unacceptable indications Contraindications	Specific indications and circumstances Other IOL indications
Near or beyond term	Women with uncomplicated pregnancies should usually be offered IOL between 41+0 and 42+0 weeks to avoid the risks of prolonged pregnancy. The exact timing should take into account the woman’s preferences and local circumstances.	IOL after 42+0/7 weeks and by 42+ 6/7 weeks is recommended (A). IOL between 41+0/7 and 42+0/7 weeks can be considered (B). See 9.	Recommended for women who are known with certainty to have reached 41 weeks (>40+7) of gestation.	Women should be offered IOL between 41+0 and 42+0 weeks, as this intervention may reduce perinatal mortality and meconium aspiration syndrome without increasing the CS rate (I–A)	For uncomplicated pregnancies, recommended after 41+0 weeks. Exact timing depends on the specific risk of stillbirth, individual preferences, and local circumstances. Waiting after 42+0 weeks is not recommended.
Gestational diabetes	Area outside of the remit of the guideline	Yes	If gestational diabetes is the only abnormality, IOL before 41 weeks of gestation is not recommended.	One of the “other indications”	One of the specific indications and circumstances
Fetal macrosomia	In the absence of any other indications, IOL should not be carried out simply because a healthcare professional suspects a baby is large for gestational age (macrosomic).	Suspected fetal macrosomia is not an indication for IOL (B) See 10	IOL at term is not recommended for suspected fetal macrosomia.	Inductions should not be performed solely for suspected fetal macrosomia. (III–D)	Not recommended on the basis of clinical suspicion of macrosomia alone.
PROM	Women with PROM at term (≥37 weeks) should be offered a choice of IOL with vaginal PGE2 or expectant management. IOL is appropriate approximately 24 hours after PROM at term.	Yes	IOL is recommended for women with PROM at term.	One of the “other indications”	For prevention of EOGBSD
Twin pregnancy	Area outside of the remit of the guideline	Not mentioned	For IOL in women with an uncomplicated twin pregnancy at or near term, no recommendation was made, as there was insufficient evidence to issue a recommendation.	Among “other indications” when uncomplicated and ≥38 weeks	In uncomplicated twin pregnancies (monochorionic or dichorionic), plan birth after 37+0 weeks.
pPROM	If a woman has pPROM, IOL should not be carried out before 34 weeks unless there are additional obstetric indications (for example, infection or fetal compromise).	At 34+0/7 weeks of gestation or greater, delivery is recommended for all women with ruptured membranes (B) See 11		One of other indications when near term with GBS negative	For prevention of EOGBSD
Previous CS	If delivery is indicated, women who have had a previous CS may be offered IOL with vaginal PGE2, CS, or expectant management on an individual basis, taking into account the woman’s circumstances and wishes.	Induction reasonable option for a maternal or fetal indication; potential increased risk of uterine rupture; avoid misoprostol See 12	Misoprostol is not recommended for IOL in women with previous CS.	Not mentioned	One of the specific indications
History of precipitate labor	IOL to avoid a birth unattended by healthcare professionals should not be routinely offered to women with a history of precipitate labor.	Included in logistic reasons for why labor may be induced	Not mentioned	One of “other indications”	Not mentioned
Maternal request	IOL should not routinely be offered on maternal request alone. However, under exceptional circumstances (for example, if the woman’s partner is soon to be posted abroad with the armed forces), induction may be considered at or after 40 weeks.	Not mentioned	Not mentioned	Unacceptable indication	Consider IOL at term based on exceptional circumstances of the woman and her family (i.e. not solely because of patient or healthcare provider preference)
Breech presentation	IOL is not generally recommended if a woman’s baby is in the breech presentation. If external cephalic version is unsuccessful, declined, or contraindicated, and the woman chooses not to have an elective CS, IOL should be offered, if delivery is indicated, after discussing the associated risks with the woman.	Not mentioned	Not mentioned	Footling breech mentioned as contraindication	Not mentioned
FGR	If there is severe FGR with confirmed fetal compromise, IOL is not recommended.	Severe FGR as possible indication	Not specifically mentioned	Suspected fetal compromise as one of the high-priority indications	For babies with FGR, use of umbilical artery, middle cerebral, and ductus venosus Doppler may assist in improving perinatal outcome through more appropriate timing of birth. Severity affects the decision concerning mode and timing of birth. If recommending expectant management, increase fetal surveillance. IOL at term to prevent stillbirth is appropriate.
IUFD	In the event of an IUFD, if the woman appears to be physically well, her membranes are intact, and there is no evidence of infection or bleeding, she should be offered a choice of immediate IOL or expectant management.	IOL is appropriate at later gestational ages (B) See 13	In the third trimester, in women with a dead or an anomalous fetus, oral or vaginal misoprostol are recommended for IOL.	One of the “other indications”	One of the specific indications
Failure of induction	Failure to establish labor after one cycle of treatment, consisting of the insertion of two vaginal PGE2 tablets (3 mg) or gel (1–2 mg) at 6-hourly intervals, or one PGE2 pessary (10 mg) within 24 hours.	Allowing at least 12–18 hours of latent labor before diagnosing a failed induction may reduce the risk of cesarean delivery	Mentioned as not necessarily indicating CS, but not specified	Not defined	The criteria for failed IOL are not generally agreed: review the individual clinical circumstances, assess fetal wellbeing using CTG, and discuss options for care. The likelihood of vaginal birth is significantly lower if not in active labor after 12 hours of oxytocin. If appropriate, consider an alternative IOL method, and/or discharge home for 24 hours followed by second attempt at IOL or CS
Assessment of the cervix	Bishop score	Bishop score	Not defined	Bishop score	Bishop score
Success of IOL	Vaginal delivery within 24 hours of IOL	Not defined	CS rate is an indicator of IOL success	Vaginal delivery within 24 to 48 hours of IOL	Not defined
Tachysystole	More than five contractions per 10 minutes for at least 20 minutes	More than five contractions in 10 minutes, averaged over a 30-minute window	Not defined	More than five contractions per 10- minute period averaged over 30 minutes.	More than five contractions in 10 minutes without fetal heart rate abnormalities
Cervical ripening	A prelude to the onset of labor whereby the cervix becomes soft and compliant. This allows its shape to change from being long and closed to being thinned out (effaced) and starting to open (dilate). It occurs either naturally or as a result of physical or pharmacological interventions.	The goal of cervical ripening is to facilitate the process of cervical softening, thinning, and dilating with resultant reduction in the rate of failed induction and induction to delivery time	Not defined	The use of pharmacological or other means to soften, efface, or dilate the cervix to increase the likelihood of a vaginal delivery.	Same as NICE
Cervical ripening timing	Vaginal PGs: maximum of two doses Vaginal PG pessary: one dose over 24 hours	Dinoprostone gel: maximum 7.5 mL within 24 hours Dinoprostone pessary	No specific mention	No maximum dose for PGs	Dinoprostone gel: maximum dose of 3 mg Dinoprostone pessary: 10 mg; second dose not recommended
Assessment of the cervix	By the Bishop score: a score of 8 or more generally indicates that the cervix is ripe	By the Bishop pelvic scoring system: an unfavorable cervix generally has been defined as a Bishop score of 6. If the total score is more than 8, the probability of vaginal delivery after labor induction is similar to that after spontaneous labor.	How can the Bishop score be used in selecting the method of IOL in clinical practice?	By the Bishop score to determine the likelihood of success and to select the appropriate method of induction. The Bishop score should be documented.	By the Bishop score (the state of the cervix is one of the important predictors of successful IOL): the cervix is unfavorable if the BS is 6 or less.

ACOG, American College of Obstetricians and Gynecologists; CS, cesarean section; CTG, cardiotocography; EOGBSD, early onset group B Streptococcal disease; FGR, fetal growth restriction; GBS, group B streptococcus; IOL, induction of labor; IUFD, intrauterine fetal death; NICE, National Institute for Health and Care Excellence; PG, prostaglandin; PGE2, prostaglandin E2 (dinoprostone); PROM, prelabor rupture of the membranes; pPROM, preterm prelabor rupture of the membranes; SCOG, Society of Obstetricians and Gynaecologists of Canada; WHO, World Health Organization.

For some precursors, there is a general consensus that recommends induction: for example, for pregnancy at or beyond term where everyone agrees on the induction between 41 ^0^ and 41 ^7^ weeks ^4– 9, 14^ or for pre-labor rupture of the membranes (PROM) ^4– 8, 11, 15^. For others, there is a general consensus that instead does not recommend induction: in the case of fetal macrosomia, all guidelines agree that macrosomia, by itself, should not be considered a precursor for induction ( Table 1 ^10^). However, a recent study that randomized 818 women, including approximately 10% diabetic patients in each group, with singleton fetuses whose estimated weight exceeded the 95th percentile showed that IOL for suspected macrosomia is associated with a reduced risk of shoulder dystocia and associated morbidity compared with expectant management, with no increase in cesarean delivery rate ^16^. The results of this study have changed the conclusions of the Cochrane review on this topic ^17, 18^.

A particular mention should be made of the so-called elective induction: the Society of Obstetricians and Gynaecologists of Canada (SOGC) ^8^ defines it as IOL in the absence of acceptable fetal or maternal indications. NICE ^4^ more generally defines "elective" clinical procedures that are planned rather than becoming necessary as emergencies and therefore applicable to other procedures as well (the most frequent being caesarean delivery). In the Queensland guidelines ^5^, elective induction seems to coincide with induction by maternal request. In truth, the concept of elective induction has increasingly been defined as an induction at term, without an apparent clinical reason, in order to improve maternal and perinatal outcomes. To my knowledge, the first reference dates back to 1947 ^19^, and since then many observational studies ^20– 28^ and some randomized studies (revised in ^29, 30^) have been published. The latest contribution is that of the Maternal–Fetal Medicine Units Network which, in August 2018 ^31^, published the results of a multicenter trial that included 6,106 low-risk women randomized to receive IOL at 39 ^0^–39 ^4^ weeks or expectant management. The primary outcome of the study was a composite of perinatal death or severe neonatal complications and the conclusions were that elective induction does not reduce the composite adverse perinatal outcome but nevertheless results in a significantly lower frequency of cesarean delivery (18.6% versus 22.2%; relative risk 0.84; 95% confidence interval [CI] 0.76 to 0.93) However, a most recent systematic review and meta-analysis of 7 randomized controlled trials with 7598, has shown no effect on the cesarean delivery rates ^32^. The rationale for elective inductions would be to manage pregnancies between 39 ^0^ and ≤41 ^0^ weeks, as before and after these gestational ages perinatal outcomes are worse than at term ^9, 33^. Overall, the results of these studies do not show clear evidence that a policy of elective induction in low-risk parturients brings a clear advantage ^29, 30^. In addition, it has been pointed out that IOL requires a range of human resources, services, monitoring, and interventions, all of which should be available if elective IOL is to be conducted safely ^34^.

Another specific mention should be made of IOL after a previous cesarean delivery: the conclusions of a recent review and meta-analysis on eight retrospective studies published between 2000 and 2013 ^35^ are that IOL slightly increases the risk of uterine rupture/dehiscence (1.1% versus 0.6%; OR 1.62; 95% CI 1.13–2.31) and of repeat cesarean delivery. However, IOL might be necessary in women with a previous cesarean delivery and should be considered a reasonable option provided that all possible measures are put in place to safeguard the well-being of mother and child ^12^.

In conclusion, for very few of the precursors reported in the guidelines ( Table 1) and in the scientific literature ^36– 41^, there is clear evidence that IOL actually improves the obstetric and/or perinatal outcome. An example for all is isolated oligohydramnios at term, a popular precursor for IOL: a recent meta-analysis ^42^ including 2,414 women with oligohydramnios and 33,585 controls but only one randomized trial with about 50 women showed that oligohydramnios represents a risk factor for IOL, caesarean section, and short-term neonatal morbidity. However, it is difficult to define precisely whether the outcomes represent the effect of IOL itself or that of the precursor. The same applies to other clinical situations in the presence of maternal and/or fetal pathology.

## Success and failure of induction of labor

These two terms would seem contradictory: if IOL succeeds, it certainly has not failed. However, even from this point of view, there is no complete uniformity in the literature. In fact, for induction success, some mean obtaining a vaginal birth, others add “not complicated”, “within 24 hours from the beginning of the induction”, or “reaching the active phase of labor” (see also Table 1). The main problem lies in the fact that success and failure in this case are not opposed: in general, the success of IOL should be represented by the achievement of a vaginal birth, even if it is operative, without a defined time limit (in other words, if the birth happens within 49 hours of the induction, does it represent an unsuccessful IOL?). This is particularly true in the case of obese women, for example, in whom it has been reported that IOL may take longer than in women of normal weight ^43^. A different issue is the failure of induction, that is the failure of the process that should have led to labor, independently from its outcome: in this sense, failure should be only one of the possible reasons why a vaginal birth is not obtained, should be related to the procedure *per se*, and should not be confused with the arrest of labor in the first or second stage once the active phase has started. However, if we look at Table 1, the uniformity between the guidelines is completely lacking: even in the case of NICE ^4^, it seems that induction failure coincides with the non-achievement of cervical ripening. It is evident that an agreement of what induction failure represents is crucial because it heavily affects the cesarean delivery rate, and many definitions have been reported ^44, 45^. In the ACOG/Society for Maternal–Fetal Medicine (SMFM) consensus on the safe prevention of the primary cesarean delivery ^46^, it is stated that “if the maternal and fetal status allow, cesarean deliveries for the induction of labor in the latent phase can be avoided by allowing longer durations of the latent phase (up to 24 hours or longer) and requiring that oxytocin be administered for at least 12–18 hours after membrane rupture before deeming the induction a failure”. Furthermore, in order to deny the definition of arrest of labor in the first stage, it is necessary to have reached at least 6 cm of dilation. This definition of failure is confirmed by the American Maternal–Fetal Medicine Units Network in a recent study of more than 10,000 women ^47^. In conclusion, it seems reasonable to say that an induction did not fail without oxytocin being administered to induce contractions.

## Prediction of successful labor induction

The identification of the factors associated with the success of the induction, intended as vaginal delivery, is fundamental for a procedure which is considered to contribute to the increase in the cesarean delivery rate ^48– 50^. One of the main factors is certainly the assessment of the cervix. From the time of its presentation ^51^, the Bishop score (BS) is the most used method to assess the cervix ^52^ ( Table 1), with a BS of 6 or less indicating an unfavorable cervix and a score of 8 or more a favorable one (and a BS of 7 being homeless). A review that considered more than 40 relatively mediocre-quality articles that correlated the BS at the beginning of the induction with its outcome concluded that BS is a poor predictor and should not be used to decide whether or not to induce labor ^53^. In an attempt to increase its predictive value, a series of clinical and biochemical parameters have been added ^54, 55^. A modified simplified BS has also been proposed which includes only dilation, station, and effacement ^56, 57^ alone or in combination with other parameters ^58^. The predictive capacity of the transvaginal sonographic assessment of the cervix has also been evaluated ^59– 61^ either by itself or in combination with other parameters ^58, 62– 66^. At present, however, the BS remains the main tool for the assessment of the cervix at the beginning of the induction and for the evaluation of cervical ripeness (i.e. its changes) during the induction process.

Other factors that have been associated with the success of the induction are parity ^67^, gestational age and size of the fetus ^68^, body mass index (BMI) ^43, 69^, age of the mother and the presence of comorbidities ^70^, and biochemical markers such as fibronectin, activin A, and insulin growth factor binding protein-1 ^48, 49, 71, 72^ either alone or variously combined ^54, 55, 63, 71, 72^. There is a general agreement in considering parity as a major predictor of IOL success ^73– 75^. Regarding the gestational age, IOL success of late preterm (34–36 ^6^ weeks) is similar to that of term pregnancies ^68^, while in weeks between 24 and 33 ^6^ it varies between 56.9 and 66.7%, considering only live births. In principle, it can be said that the success of the induction, meaning vaginal birth, increases with the gestational age and that >50% of women (also nulliparous with an unripe cervix) give birth vaginally ^76^. Scoring systems ^73^, nomograms ^74^, and prediction model systems ^75^ have been proposed but have not been validated yet ^77^. Recently, a systematic review of 14 models derived or validated since 1966 has provided a list of recommendations for improving the performance and utilization of the models ^78^.

## Methods of induction

The literature concerning the various methods of labor induction (i.e. cervical ripening and the onset of uterine contractions) discusses the effectiveness of pharmacological, mechanical, investigational, and complementary and alternative medicine means of third trimester IOL ^79– 84^. The pharmacological methods are oxytocin and prostaglandins (PGE1: misoprostol and PGE2: dinoprostone), the latter available in different forms (tablet, gel, or insert) and, for misoprostol, with different routes of administration (oral titrated solution, buccal/sublingual, oral, or vaginal) ^84, 85^. Slow-release formulations are also available for PGEs. In general, PGEs are the drug of choice when cervical ripening is needed in the presence of an unfavorable cervix (see Table 1). Cervical ripening can be performed in either an outpatient or an inpatient setting ^86– 88^. Oxytocin, instead, is used when the cervix is favorable (BS 7–8) and is the drug that induces contractions. Generally speaking, it could be correct to state that an IOL, in the presence of maternal and fetal well-being, should not be defined failed before oxytocin administration. In other words, NICE’s definition ^4^ of failure of the induction appears, according to modern knowledge, difficult to share. Suffice it to say that IOL was widespread practice even before the introduction of PGEs and that at the time it was based only on the administration of oxytocin, with good results ^19^. Among the mechanical methods, the most popular is the Foley catheter ^89, 90^, either alone ^91^ or in association with oxytocin ^92– 96^, or misoprostol ^97, 98^, and with different balloon volumes ^99, 100^. The combination of mechanical and pharmacological methods used simultaneously does not show clear benefits in terms of mode of delivery: the use of the Foley catheter with oxytocin increases the rate of delivery within 24 hours in nulliparas ^95, 96^, while the association of Foley and misoprostol ^101^ reduces the intervention to delivery time interval and the number of uterine hyperstimulations, in both cases without influencing cesarean delivery rates.

Alternative methods include castor oil, which has received renewed interest in recent times ^102– 104^, acupuncture ^105^, breast/nipple stimulation ^106– 108^, sexual intercourse ^109^, homeopathy ^110^, and hypnotic relaxation ^111^. For all these methods, the role in IOL is uncertain, basically because of the lack of studies, if not anecdotal reports. Membrane sweeping deserves a special mention: in spite of modest discomfort for the mother, it reduces the number of pregnancies beyond term and the need for induction, without increasing the infectious risks ^112^. All the cited guidelines recommend its execution in all women starting from 40 ^0^ weeks to reduce the incidence of IOL and also before the pharmacological IOL ^4– 8^. If the cervix is closed and membrane sweeping is not possible, cervical massage in vaginal fornices may achieve similar effect ^5^.

I do not feel able to state favor for one method of cervical ripening over another: often the choice of the drug to be used also passes through local policies, but this is beyond the scope of this review. However, consensus seems to be unanimous that fetal heart rate should be recorded both before and after cervical ripening, intracervical prostaglandins can be abandoned, and misoprostol should be avoided in the induction of women with a previous cesarean delivery.

## Conclusions

The purpose of this review is to give some “food for thought”, showing that the variables involved in the process are many and ideally should be evaluated on a case-by-case basis. The attempts made to create successful prediction systems implemented so far are still far from achieving the intended. In conclusion, some suggestions can be provided: it would be recommended that every birth center should be provided with local guidelines regarding the IOL; once started, IOL should be continued until the end; there is no evidence that repeated cycles of cervical ripening are advantageous in terms of successful induction (and unfortunately the birth experience in women whose pre-induction process was eternal has not yet been investigated thoroughly); and the lack of changes of the BS at the end of cervical ripening is not synonymous with IOL failure. It has been reported that even in nulliparous women with an unfavorable BS, unchanged after the ripening process with PGEs, the administration of oxytocin leads to 80% of vaginal deliveries ^66^.
